# Geographical heterogeneity and influenza infection within households

**DOI:** 10.1186/1471-2334-14-369

**Published:** 2014-07-03

**Authors:** Day-Yu Chao, Kuang-Fu Cheng, Ying-Hen Hsieh, Tsai-Chung Li, Trong-Neng Wu, Chiu-Ying Chen

**Affiliations:** 1 Graduate Institute of Microbiology and Public Health, College of Veterinary Medicine, National Chung-Hsing University, Taichung, Taiwan; 2 School of Public Health and Biostatistics Center, Taipei Medical University, Taipei, Taiwan; 3 Graduate Institute of Biostatistics, China Medical University, Taichung, Taiwan; 4 Department of Public Health, China Medical University, Taichung, Taiwan

**Keywords:** Influenza, Trivalent Inactivated Vaccine (TIV), Children, Household contacts, Geographical heterogeneity

## Abstract

**Background:**

Although it has been suggested that schoolchildren vaccination reduces influenza morbidity and mortality in the community, it is unknown whether geographical heterogeneity would affect vaccine effectiveness.

**Methods:**

A 3-year prospective, non-randomized sero-epidemiological study was conducted during 2008–2011 by recruiting schoolchildren from both urban and rural areas. Respective totals of 124, 206, and 176 households were recruited and their household contacts were followed. Serum samples were collected pre-vaccination, one-month post-vaccination and post-season from children and household contacts for hemagglutination inhibition (HI) assay. A multivariate logistic model implemented with generalized estimation equations (GEE) was fitted with morbidity or a four-fold increase in HI titer of the household contacts for two consecutive sera as the dependent variable; with geographical location, vaccination status of each household and previous vaccination history as predictor variables.

**Results:**

Although our results show no significant reduction in the proportion of infection or clinical morbidity among household contacts, a higher risk of infection, indicated by odds ratio > 1, was consistently observed among household children contacts from the un-vaccinated households after adjusting for confounding variables. Interestingly, a statistically significant lower risk of infection was observed among household adult contacts from rural area when compared to those from urban area (OR = 0.89; 95% CI: 0.82-0.97 for Year 2 and OR = 0.85; 95% CI: 0.75-0.96 for Year 3).

**Conclusions:**

A significant difference in the risk of influenza infection among household adults due to geographical heterogeneity, independent of schoolchildren vaccination status, was revealed in this study. Its impact on vaccine effectiveness requires further study.

## Background

Influenza is a major cause of morbidity and mortality, resulting in an estimated 3–5 million cases of severe influenza illness annually [1]. Although older adults have the highest influenza-related mortality, children who have contracted influenza infection experience substantial morbidity, resulting in absence from school, extra working days for parents and increased health care costs from purchasing antibiotics [2-4]. Additionally, children attending day-care centers and elementary schools have long been identified as the major causes of influenza virus transmission in the community since they can shed greater amounts of virus for longer periods of time [5-8]. In fact, the best predictor for influenza occurring in a household is the presence of children [6]. Focusing efforts for influenza vaccination on school-aged children may therefore be an effective and practical method for reducing the burden of influenza in the community. Since 2008, the Advisory Committee on Immunization Practices (ACIP) of the Centers for Disease Control and Prevention (CDC) in the U.S.A. expanded universal influenza vaccination recommendations to all children aged 0.5-18 years [9].

A systematic review suggested that although evidence exists that vaccinating healthy children has the potential of reducing the effect of influenza transmission within households and community, further data are needed because of limitations in study design, varied vaccination policy implemented by different countries and prior experience of receiving vaccination which make the benefits from vaccination difficult to quantify [10-14]. Furthermore, recent studies from 2009 pandemic H1N1 (pH1N1) outbreaks suggest that transmissions of influenza virus were spatially heterogeneous [15]. So far, it is unknown whether spatially heterogeneous transmission of influenza virus affects the infection rate and vaccine effectiveness within households.

Since 2007, the government in Taiwan has implemented a free vaccination program through which all schoolchildren in grades 1–4 receive a free annual trivalent influenza vaccination (TIV), which later expanded to grades 1–6 after 2010: a single dose from commercially-available TIV containing 0.5-mL of 15ug HA of the H1N1, H3N2 and B antigen would be administered through a school-based delivery program. From 2008–2011, we recruited elementary schoolchildren from both urban and rural areas during three consecutive influenza seasons and followed up three different cohorts of their household contacts. The current non-randomized study offered a unique opportunity to evaluate (1) the effect of vaccinating school-aged children on reducing virus transmission and influenza related morbidity among their household contacts, and (2) the influence of geographical location on acquiring influenza infection among the household contacts.

## Methods

### Subject enrollment and serological specimens

**Figure 1:**
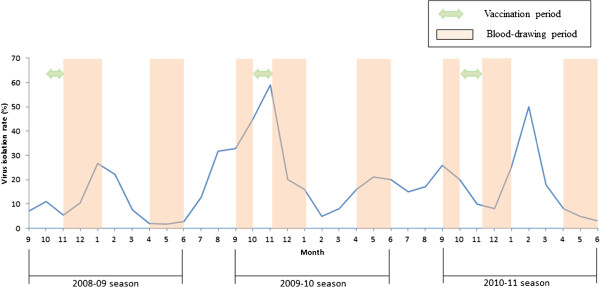
Timeframe of TIV vaccination, blood collection as well as influenza virus isolation rates from 2008 to 2011.

During three consecutive influenza seasons from 2008 to 2011, a prospective non-randomized sero-epidemiological study was conducted by the influenza research group at National Chung-Hsing University (NCHU) and China Medical University (CMU) to investigate household transmission and vaccine effectiveness. Students from two urban (Taichung City) elementary schools and four rural (Nantou County) schools in central Taiwan were recruited for this three-year study starting in the fall of 2008. Taichung city is the largest urban city in central Taiwan with a population of more than 1 million and has a highly developed socioeconomic structure. The two schools selected were located respectively in the North and Central districts of the city, with approximately 140 thousand total residents. The nearby Nantou County, which has a total land size approximately 25 times larger than that of Taipei City, is the second largest county and the only landlocked county on the island of Taiwan with a population of over 500 thousand, and is comparatively less developed socioeconomically. The four schools in Nantou County were selected purposely from four different administrative districts in the county; namely, Nantou City and Tsaotun Township each with around 100 thousand residents, and the rural townships of, Mingjian, and Guoshing with 40 and 20 thousand residents, respectively. Since the study protocol involved intensive visiting and blood-drawing, all children from the selected schools of grades 1–4 during the first and second study year and grades 1–6 during the third study year were given a detailed description of the study protocol and a returned slip was included to acknowledge if they were willing to participate in the study voluntarily. Each successive year, some families dropped out and additional volunteer subjects were recruited. Family members of the school-aged children were also recruited to join the study to further understand vaccine effectiveness in preventing household transmissions of seasonal influenza viruses. The serum samples including pre-vaccination (collected between September and October), one month post-vaccination (collected between November and January) and post-season (collected between April and June) were taken from children participants by trained nurses, except during the 2008–2009 season when only sera from post-vaccination and post-season periods were taken as shown in Figure 1. Since TIV was not free for household adult contacts unless they were aged greater than 65 years and the proportion of TIV vaccination was less than 0.1%, sera were taken from adult contacts only during pre- and post-vaccination during the three consecutive seasons. All subjects gave informed consent and the study was approved by the Medical Ethics Committee of China Medical University (DMR96-IRB-216). The study protocol based on laboratory data was established to assess the serological responses by including the paired sera drawn from two consecutive time periods, in order to define immune response before and after vaccination or before vaccination and post-season among household contacts. The obtained sera were evaluated for antibody titers by measuring a hemagglutination inhibition (HI) assay following the standard protocol by the World Health Organization (WHO) using the contemporary vaccine and wild-type strains of influenza viruses as described in the laboratory methods section below.

### Data collection

All study subjects including children participants and household contacts were assessed for signs and symptoms of influenza throughout the follow-up period, defined by the start date (>1 laboratory-confirmed influenza cases in 2 consecutive weeks from sentinel sites) and termination date (no laboratory-confirmed influenza cases for 2 consecutive weeks from sentinel sites) based on the influenza surveillance network coordinated by Taiwan-CDC which covers approximately 75% of the basic administrative units of Taiwan (cities or districts) and routinely collects clinical specimens [16,17]. The average influenza virus isolation rates per month during three consecutive seasons were shown in Figure 1. During each influenza season, two questionnaires were also administered by trained interviewers regarding basic demographic and social contact information and whether a seasonal influenza vaccination had been received in the past and present years. Information regarding comorbidities including cardiovascular disease, hypertension, or diabetes mellitus was also taken from the adults in the family. Bi-weekly clinical symptom reviews were carried out using a standardized questionnaire via a telephone interview between October and June. Participants were asked to report any newly experienced febrile respiratory symptoms, including fevers (≥38°C), sore throats, cough, nasal congestion, headache, sinus problems, muscle aches, fatigue, ear ache or infection or chills during the influenza season. The overall completion rate for the questionnaires was more than 90%.

### Laboratory methods

Antibody titers were measured using HI assay which adhered to the WHO protocol [18,19]. In brief, serum samples were pre-treated with receptor destroying enzyme (RDE, Deka Seriken Co Ltd, Tokyo, Japan) in 1:4 ratio at 37°C for 16 hours, followed by another 30 minutes at 56°C and then an equal volume of 1.6% trisodium citrate was added for enzyme inactivation. The different strains of influenza viruses used in this study were first prepared from the culture supernatants of infected Madin-Darby canine kidney (MDCK) cells. Twenty-five microliters (μl) (4 hemagglutination units, HA) of influenza virus were incubated at room temperature for one hour with an equal volume of RDE-treated serum in a V-shape 96-well microtiter plate. After incubation, 25 μl of 1% (vol/vol) chicken red blood cells was added to each well. Hemagglutination inhibition was read after 30 minutes. To evaluate asymptomatic infection, influenza viruses from the contemporary vaccine and wild-type strains which represented more than 80% of circulating viruses during the three consecutive influenza seasons were used. Details are listed in Table 1. For the HI assay, serum samples were tested with an initial dilution of 1:10 and a final dilution of 1:10,240 and the titers were expressed as the reciprocal of the highest dilution of serum where hemagglutination was prevented. Samples with dilution higher than 10,240 where hemagglutination was prevented were repeated to obtain the final HI titers. Samples that were negative by HI were assigned a titer of 1:5 for computational purposes in obtaining a geometric mean titer (GMT) or seroconversion rate. We defined seroconversion as a four-fold or greater rise of HI antibody titers to influenza A (H1N1), A (H3N2) and B to assess serological evidence of viral infection among household contacts. In this study, we excluded household contacts who had TIV vaccination during the influenza seasons in the analysis to avoid false classification. This allows us to use a four-fold increase in the sera between pre- and post-season as a surrogate marker for viral infection, and compare the likelihood of infection among household contacts between households of different vaccination statuses as later described in the statistical analysis section.

**Table 1:** The contemporary vaccine and wild-type strains circulated during the three consecutive influenza seasons used in this study

**Strain**	**2008-2009(1st Year)**	**2009-2010(2nd Year)**	**2010-2011(3rd Year)**
Vaccine strain			
H1N1	A/Brisbane/59/2007	A/Brisbane/59/2007	
pdmH1N12009^2^		A/California/07/2009	A/California/07/2009
H3N2	A/Brisbane/10/2007	A/Brisbane/10/2007	A/Perth/16/2009
flu B	B/Florida/4/2006(Y)^1^	B/Brisbane/60/2008(V)^1^	B/Brisbane/60/2008(V)
Wildtype strain			
H1N1	A/Taiwan/606/2008		
pdmH1N12009^2^		A/California/07/2009	A/Taiwan/5506/2010
			A/Taiwan/5520/2011
H3N2	A/Taiwan/736/2008	A/Taiwan/480/2008	A/Taiwan/3869/2010
	A/Taiwan/480/2008	A/Taiwan/736/2008	A/Taiwan/3814/2011
		A/Taiwan/3982/2009	
flu B	B/Taiwan/29/2008(Y)	B/Taiwan/5908/2009(V)	B/Taiwan/3591/2010(V)
			B/Taiwan/5806/2011(Y)

^1^Y refer to Yamagada lineage and V refer to Victoria lineage.

^2^pdmH1N12009: pandemic H1N1 in 2009.

### Statistical analysis

Vaccination status of each household was categorized as  complete’ ,  partial’ or  non-vaccination’ based on the vaccination status of its occupying school-aged child/children (named as participants). If all participants in the household received or did not receive TIV during the season, the household was classified as  complete’ or  un-vaccination’ , respectively. If only some of the participants from the household received TIV during the season, the household was classified as  partial’. To estimate vaccine effectiveness in preventing respiratory illness or infection among household contacts, a logistic model was fitted with the illness status or four-fold increase of HI titer during two consecutive sera of the household contacts as the dependent variable and the vaccination status of each household as the predictor variable. Generalized estimation equations (GEE) method was used to account for correlation among members of the same household. We tested the hypothesis that there was no difference in the rate of respiratory illnesses or related infection among the household contacts of completely vaccinated, partially vaccinated and un-vaccinated households. All statistical analyses were conducted using SAS (release 9.2, SAS Institute) software. A p-value <0.05 was considered statistically significant.

## Results

### Subject recruitment and demographics

**Figure 2:**
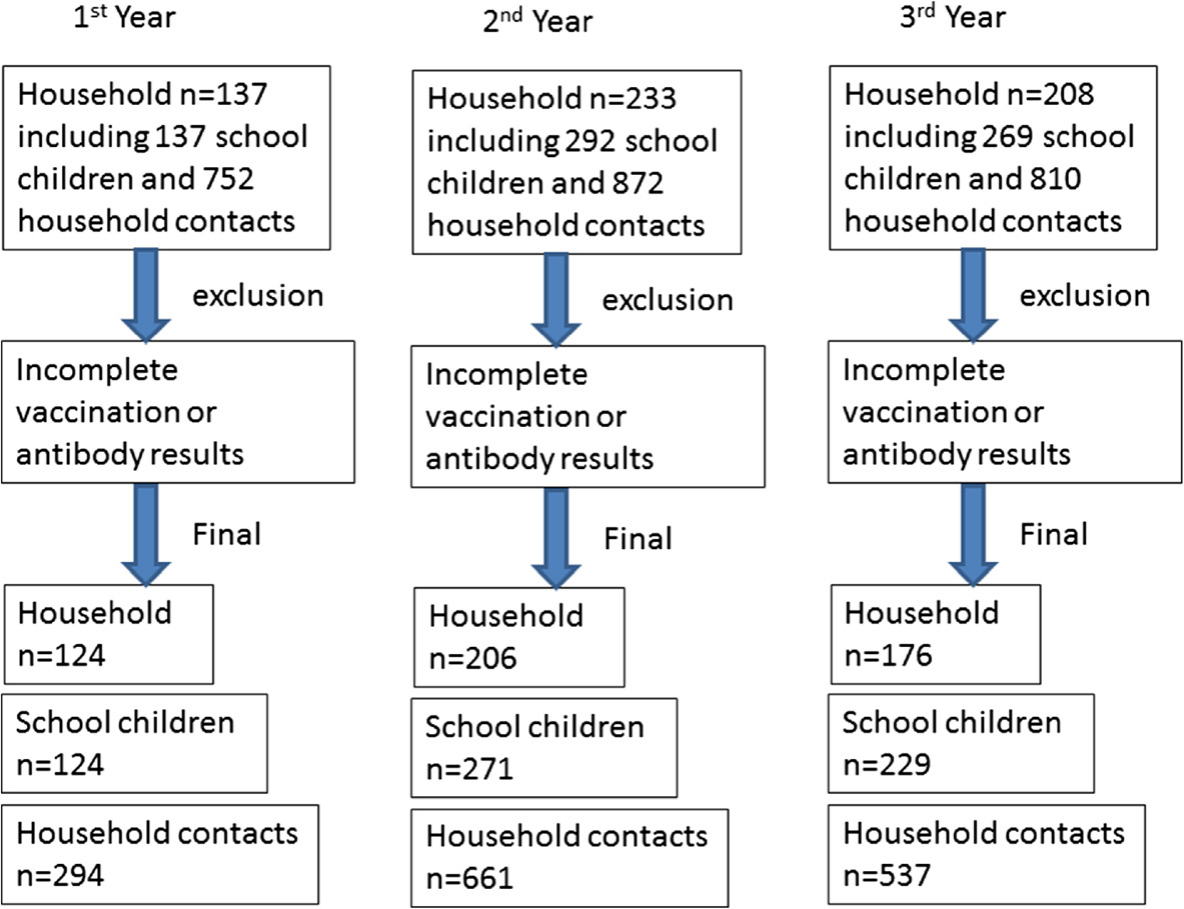
Flow chart of participant recruitment for all three years.

Figure 2 describes the numbers of participants and household contacts recruited in this study during the three consecutive years and the final numbers entered for statistical analysis. Except for the first year, there was more than one school-aged child included in the study from each household.

For households considered in the final analysis, we stratified them into  complete’ ,  partial’ and  un-vaccinated’ according to the immunization status of the participants from each household as previously described. Table 2 shows the basic demographics of participants and household contacts from three consecutive seasons. The household sizes were about the same across all three consecutive seasons - the partially-immunized households were slightly larger but not significantly different from the un-vaccinated or completely-vaccinated households. A higher proportion of recruited participants from the urban area (Taichung) did not receive TIV (TIV(-) families) except in Year 2 (51% from TIV(+) vs 30% from TIV(-) and 43% from partial TIV families), probably due to the occurrence of pandemic H1N1 (pH1N1) in 2009.

**Table 2:** Demographic characteristics of trivalent influenza vaccine (TIV) completed, partial and non-vaccinated households during the three consecutive years of influenza seasons (2008–2011)

	**1st Year**			**2nd Year**				**3rd Year**			
	**TIV(-)**	**TIV(+)**	**p**	**TIV(-)**	**Partial TIV**	**TIV(+)**	**p**	**TIV(-)**	**Partial TIV**	**TIV(+)**	**p**
**No of household**											
Household size^#^	3.28	3.45		4.2	7.46	3.84		3.76	7.50	4.12	
	(3.00,3.58)	(3.18,3.72)		(3.83,4.64)	(6.51,8.40)	(3.35,4.34)		(3.15,4.36)	(6.06,8.94)	(3.70,4.54)	
Location of residence											
Taichung	28(0.47)	22(0.38)		34(0.30)	15(0.43)	29(0.51)	*	20(0.49)	9(0.45)	35(0.30)	*
Nantou	31(0.53)	43(0.62)		80(0.70)	20(0.57)	28(0.49)		21(0.51)	11(0.55)	80(0.70)	
**Participants**											
Gender											
Male	31(0.53)	34(0.52)		71(0.52)	33(0.47)	31(0.48)		19(0.41)	21(0.49)	75(0.54)	
Female	28(0.47)	31(0.48)		66(0.48)	37(0.53)	33(0.52)		27(0.59)	22(0.51)	65(0.46)	
Age (average years)^#^	9.24	8.00	*	9.39	9.20	9.11		10.80	9.65	9.66	*
	(8.83,9.64)	(7.64,8.36)		(9.10,9.68)	(8.75,9.65)	(8.76,9.45)		(10.24,11.37)	(9.04,10.26)	(9.39,9.94)	
**Household contacts**											
Gender											
Male	60(0.44)	63(0.40)		152(0.44)	78(0.44)	55(0.40)		40(0.37)	45(0.42)	142(0.44)	
Female	75(0.56)	96(0.60)		192(0.56)	100(0.56)	84(0.60)		68(0.63)	61(0.58)	181(0.56)	
Age											
<18	37(0.27)	43(0.27)		80(0.23)	45(0.25)	15(0.11)	*	31(0.29)	32(0.30)	74(0.23)	
≥18	98(0.73)	116(0.73)		264(0.77)	133(0.75)	124(0.89)		77(0.71)	74(0.70)	249(0.77)	
Age (average years)^#^	34.35	33.51		36.84	36.19	40.22		35.47	31.81	36.49	
	(31.46,37.24)	(30.82,36.20)		(34.91,38.77)	(33.51,38.87)	(37.58,42.85)		(31.79,39.16)	(28.63,34.99)	(34.55,38.43)	
Vaccinated in previous year											
Yes	9(0.07)	29(0.18)		7(0.02)	21(0.12)	23(0.17)	*	16(0.15)	18(0.17)	68(0.21)	
No	126(0.93)	130(0.82)		337(0.98)	157(0.88)	116(0.83)		92(0.85)	88(0.83)	255(0.79)	

*p < 0.05.

^#^Numbers in brackets are 95 % confidence interval calculated by GEE method.

For children participants from each household, no gender difference was found during all three years. However, it was found that during Years 1 and 3, children participants who received TIV vaccination were slightly younger compared to those who did not. For household contacts, a significantly higher proportion of young contacts (age less than 18 years) was found in un-vaccinated (23%) and partially-vaccinated (25%) groups than from the completely-vaccinated group (11%) during the second influenza season (p < 0.05). A significantly higher proportion of household contacts who received TIV during the previous season was also found in  complete’ and  partial’ vaccination households than from  un-vaccination’ households during the first and second year of study (p < 0.05) (Table 2).

### Immune response and vaccine efficacy among schoolchildren participants

Table 3 shows the antibody titers from the schoolchildren participants pre- and post-vaccination during three consecutive influenza seasons. A significantly higher antibody titer of post-vaccination sera in participants who received TIV vaccination, compared to the titer in participants without receiving TIV, was observed during Year 1 and 3. Meanwhile, significantly higher immune responses, including GMT and proportion of HI equal to or greater than 40, were observed among participants receiving TIV than those without (Table 3). Higher proportions of participants with 4-fold increase of antibody titer were also observed among the group receiving TIV than the group without (51% vs 16% for H1N1 and 16% vs 0% for B antigens). No difference in antibody titers was observed between TIV(+) and TIV(-) participants in the post-season sera for all three study years.

**Table 3:** Hemagglutination antibody titers of schoolchildren participant sera obtained before, after vaccination and post-season during three consecutive years

	**1st Year**			**2nd Year**			**3rd Year**		
	**TIV(-)**	**TIV(+)**	**P**	**TIV(-)**	**TIV(+)**	**P**	**TIV(-)**	**TIV(+)**	**P**
	**N = 59**	**N = 65**		**N = 172**	**N = 99**		**N = 67**	**N = 162**	
**Pre-vaccination**									
**HI-titer**≧40									
H1N1	-	-		155(0.90)	91(0.92)		45(0.67)	124(0.77)	
H3N2	-	-		142(0.83)	77(0.78)		17(0.25)	58(0.36)	
B	-	-		44(0.26)	26(0.26)		49(0.73)	104(0.64)	*
**GMT**^**#**^									
H1N1	-	-		263.78(211.80,315.75)	412.83(246.23,579.42)	*	82.76(60.50,105.02)	89.38(75.14,103.62)	
H3N2	-	-		210.26(168.06,252.46)	245.91(166.90,324.92)		52.54(20.37,84.70)	85.40(57.74,113.06)	
B	-	-		25.76(21.17,30.34)	30.81(22.25,39.36)		69.18(53.71,84.65)	75.43(54.95,95.92)	
**Post-vaccination**									
**HI-titer**≧40									
H1N1	33(0.56)	60(0.92)	*	112(0.65)	61(0.62)		37(0.55)	132(0.81)	*
H3N2	51(0.86)	59(0.91)		114(0.66)	61(0.62)		6(0.09)	49(0.30)	*
B	26(0.44)	43(0.66)	*	72(0.42)	39(0.39)		23(0.34)	73(0.45)	
**GMT**^**#**^									
H1N1	161.80(91.12,232.48)	422.77(232.82,612.71)	*	408.08(306.32,509.84)	324.14(245.96,402.32)		97.76(19.45,176.07)	121.33(100.60,142.06)	
H3N2	353.93(253.05,454.81)	411.41(312.73,510.09)		487.59(407.64,567.53)	455.61(368.75,542.46)		20.82(12.91,28.73)	61.82(41.07,82.57)	*
B	115.74(67.21,164.28)	114.24(83.34,145.14)		46.48(38.09,54.87)	48.59(36.73,60.45)		33.13(24.06,42.21)	44.26(33.37,55.15)	
**4-Fold increase (Post vs Pre-vaccination)**									
H1N1	-	-		39(0.23)	17(0.17)		11(0.16)	82(0.51)	*
H3N2	-	-		60(0.35)	36(0.36)		3(0.04)	15(0.09)	
B	-	-		62(0.36)	31(0.31)		0(0.00)	26(0.16)	*
**Post-season**									
**HI-titer≧40**									
H1N1	40(0.68)	60(0.68)		154(0.90)	89(0.90)		45(0.67)	116(0.72)	
H3N2	48(0.82)	59(0.82)		163(0.95)	95(0.96)		28(0.42)	75(0.46)	
B	24(0.40)	43(0.40)		167(0.97)	93(0.94)		26(0.39)	68(0.42)	
**GMT**^**#**^	-	-							
H1N1	129.26(71.17,187.34)	157.46(68.66,246.25)		570.61(497.40,643.82)	503.08(410.42,595.74)		132.69(87.48,177.89)	90.52(77.27,103.78)	*
H3N2	272.46(159.43,385.48)	209.03(120.55,297.52)		319.04(269.30,368.79)	390.61(316.43,464.78)		99.63(34.88,164.38)	165.77(110.13,221.41)	
B	40.43(28.70,52.15)	55.53(38.39,72.67)		23.46(20.42,26.50)	24.70(20.09,29.31)		31.34(22.69,40.00)	37.87(28.82,46.92)	

*p < 0.05.

^#^Numbers in brackets are 95% confidence interval calculated by GEE method.

Since pH1N1 circulated before and during the second influenza year, TIV vaccine was delivered one month ahead of pH1N1 vaccination. We further divided the participants from Year 2 with or without receiving TIV into four groups based on their status of receiving pH1N1 vaccination. As shown in Table 4, a significantly higher proportion of participants having HI titer greater than or equal to 40 in the groups receiving the pH1N1 vaccine was observed than in the group without receiving the pH1N1 vaccine, particularly among the groups receiving the pH1N1 but not TIV vaccine (p < 0.05). Similar results were observed when comparing GMT titers between two groups. Interestingly, higher antibody titers against seasonal influenza strains including H1, H3 and B were observed in children who received the pH1N1 vaccine than in children without the pH1N1 vaccine regardless of their TIV history (Table 4).

**Table 4:** Hemagglutination antibody titers during the second influenza season based on TIV or pH1N1 vaccination status of schoolchildren participants

**Participants(schoolchildren)**	**TIV(-) N = 172**			**TIV(+) N = 99**		
	**pH1N1(-) N = 17**	**pH1N1(+) N = 155**	**P**	**pH1N1(-) N = 6**	**pH1N1(+) N = 93**	**P**
**Pre-vaccination**						
**HI-titer**≧40						
pH1N1	11(0.65)	112(0.72)		4(0.67)	68(0.73)	
H1N1	14(0.82)	141(0.91)		4(0.67)	87(0.94)	
H3N2	13(0.77)	129(0.83)		5(0.83)	72(0.77)	*
B	5(0.29)	39(0.25)		2(0.33)	24(0.26)	
**GMT**^**#**^						
pH1N1	48.24(28.72,67.75)	56.77(49.45,64.10)		65.00(7.23,122.77)	57.58(48.92,66.24)	
H1N1	162.94(79.96,245.92)	274.84(217.97,331.71)	*	433.33(-64.89,931.55)	411.51(235.43,587.58)	
H3N2	207.06(42.89,371.22)	210.61(166.67,254.56)		107.50(40.65,174.35)	254.84(171.02,338.65)	*
B	17.65(9.77,25.52)	26.65(21.64,31.65)	*	66.67(-64.31,197.64)	28.49(21.90,35.09)	
**Post-vaccination**						
**HI-titer**≧40						
pH1N1	2(0.12)	101(0.65)	*	2(0.33)	52(0.56)	*
H1N1	4(0.24)	108(0.70)	*	3(0.50)	58(0.62)	*
H3N2	4(0.24)	110(0.71)	*	4(0.67)	57(0.61)	
B	2(0.12)	70(0.45)	*	3(0.50)	36(0.39)	
**GMT**^**#**^						
H1N1_p	49.41(30.22,68.60)	216.65(167.73,265.56)	*	84.17(14.68,153.65)	228.60(154.87,302.33)	*
H1N1	202.94(40.19,365.69)	430.58(319.28,541.88)	*	270.00(45.61,494.39)	327.63(245.00,410.27)	
H3N2	220.00(58.35,381.65)	516.94(430.86,603.01)	*	213.33(-9.85,436.51)	471.24(380.18,562.29)	*
B	23.24(10.48,35.99)	49.03(39.89,58.17)	*	148.33(-3.62,300.29)	42.15(33.09,51.21)	

*p < 0.05.

^#^numbers in brackets are 95% confidence interval calculated by GEE method.

### Effectiveness of vaccination of schoolchildren in reducing illness or serologically proven infection among household contacts

Previous studies suggested that schoolchildren vaccinated with TIV could reduce household and community transmission of influenza virus [10-14]. Our results did not detect significant reduction in the proportion of infection or clinical morbidity among contacts of the households with complete TIV vaccination compared to those with partial or no vaccination (Table 5). Also, we further divided the household based on the status of receiving TIV or pH1N1 vaccines during the 2009–2010 influenza season due to the pH1N1 epidemic. Because of the low numbers of the households did not receive the pH1N1 vaccine, infection rates of contacts from Year 2 were removed. Higher infection rates (38% of type H3 and 84% of any type) were observed among household adult contacts from families receiving both TIV and pH1N1 vaccines than from families not receiving TIV (26% of type H3 and 76% of any type) with statistical significance (p < 0.05) (Table 5). Similar results were observed among household children contacts.

**Table 5:** Proportions of infection or clinical morbidity among household contacts of children and adults among three TIV vaccination status groups during the three consecutive influenza seasons

	**1st Year**			**2nd Year**				**3rd Year**			
**Children, Number (proportion)**	**TIV(-)**	**TIV(+)**	**P**	**TIV(-)**	**Partial TIV**	**TIV(+)**	**P**	**TIV(-)**	**Partial TIV**	**TIV(+)**	**P**
	**N = 37**	**N = 43**		**N = 72**	**N = 38**	**N = 14**		**N = 31**	**N = 32**	**N = 74**	
**4-fold increase**											
H1N1	6(0.16)	6(0.14)		29(0.40)	10(0.26)	3(0.21)		13(0.42)	12(0.38)	31(0.42)	
H3N2	4(0.11)	14(0.33)		30(0.42)	17(0.45)	4(0.29)		11(0.35)	6(0.19)	26(0.35)	
B	4(0.11)	3(0.07)		35(0.49)	17(0.45)	7(0.50)		6(0.19)	14(0.44)	18(0.24)	
pH1N1	-	-		55(0.76)	27(0.71)	13(0.93)		-	-	-	
Any 4-fold increase	14(0.38)	20(0.47)		44(0.61)	23(0.61)	7(0.50)		22(0.71)	21(0.66)	52(0.70)	
**Symptoms**											
Any	17(0.46)	29(0.67)		36(0.50)	22(0.58)	9(0.64)		19(0.61)	13(0.41)	37(0.50)	
Fever	5(0.14)	14(0.33)		7(0.10)	1(0.03)	1(0.07)		3(0.10)	6(0.19)	9(0.12)	
Fever plus any two symptoms	3(0.08)	10(0.23)		4(0.06)	0(0.00)	0(0.00)		1(0.03)	4(0.13)	7(0.09)	
**Any 4-fold increase plus any symptoms**	5(0.14)	15(0.35)	*	24(0.33)	13(0.34)	5(0.36)		27(0.87)	24(0.75)	62(0.84)	
**Adults, Number (proportion)**	**TIV(-)**	**TIV(+)**	**P**	**TIV(-)**	**Partial TIV**	**TIV(+)**	**P**	**TIV(-)**	**Partial TIV**	**TIV(+)**	**P**
	**N = 98**	**N = 116**		**N = 222**	**N = 111**	**N = 109**		**N = 77**	**N = 74**	**N = 249**	
**Any 4-fold increase**											
H1N1	0(0.00)	2(0.02)		70(0.32)	46(0.41)	41(0.38)		10(0.13)	19(0.26)	60(0.24)	
H3N2	9(0.09)	17(0.15)		57(0.26)	38(0.34)	41(0.38)	*	3(0.04)	0(0.00)	6(0.02)	
B	5(0.05)	3(0.03)		70(0.32)	45(0.41)	38(0.35)		4(0.05)	6(0.08)	18(0.07)	
pH1N1	-	-		109(0.49)	57(0.51)	55(0.50)		-	-	-	
Any 4-fold increase	14(0.14)	20(0.17)		135(0.61)	74(0.67)	75(0.69)		11(0.14)	20(0.27)	64(0.26)	
**Symptoms**											
Any	43(0.44)	52(0.45)		55(0.25)	34(0.31)	39(0.36)	*	30(0.39)	31(0.42)	105(0.42)	
Fever	9(0.09)	5(0.04)		1(0.00)	4(0.04)	2(0.02)		2(0.03)	1(0.01)	7(0.03)	
Fever plus any two symptoms	0(0.00)	1(0.01)		0(0.00)	0(0.00)	0(0.00)		1(0.01)	0(0.00)	5(0.02)	
**Any 4-fold increase plus any symptoms**	7(0.07)	7(0.06)		39(0.18)	24(0.22)	24(0.22)		38(0.49)	42(0.57)	131(0.53)	

*P < 0.05.

To further investigate if the proportion of serological infection or clinical morbidity of household contacts was associated with other confounding variables, multivariate analysis using GEE was implemented to adjust for variables including underlying diseases, household size, residential location, TIV during the previous year, the status of household receiving TIV. The results were shown in Table 6. Gender of household contacts was not included due to the lack of difference shown between the comparison groups in Table 2. Although mostly insignificant, a higher risk of infection, indicated by odds ratio greater than 1, was consistently observed among household children contacts from the un-vaccinated households. During the first year, a significantly higher risk of infection and clinical morbidity was observed among children contacts from un-vaccinated households than from complete vaccinated households (p < 0.05). No statistical significance for risk of infection and morbidity was observed among household adult contacts and odds ratios were not consistently greater than 1.

**Table 6:** Multivariate logistic regression on proportion of infection or plus clinical morbidity among household contacts during three consecutive influenza seasons

		**Any 4-fold increase**^**$**^	**p**	**Any 4-fold increase plus any symptoms**^**$**^	**p**
Children					
**1st Year**	Rural v.s urban	1.03 (0.79,1.34)	0.835	0.93 (0.76,1.13)	0.458
	Household size	0.97 (0.85,1.11)	0.692	0.96 (0.87,1.06)	0.385
	TIV previous year	0.72 (0.42,1.22)	0.221	0.97 (0.76,1.25)	0.831
	TIV (-) v.s (+)	1.26 (0.93,1.70)	0.139	1.27 (1.03,1.56)	0.024*
**2nd Year**	Rural v.s urban	0.95 (0.79,1.13)	0.547	0.93 (0.76,1.14)	0.505
	Household size	1.01 (0.81,1.27)	0.901	1.00 (0.79,1.27)	0.997
	TIV previous year	0.91 (0.60,1.38)	0.664	1.10 (0.70,1.75)	0.672
	TIV (-) v.s (+)	1.08 (0.76,1.52)	0.664	1.03 (0.70,1.52)	0.886
	TIV partial v.s (+)	1.09 (0.75,1.58)	0.665	1.06 (0.68,1.66)	0.800
**3rd Year**	Rural v.s urban	1.10 (0.96,1.25)	0.171	1.02 (0.84,1.24)	0.834
	Household size	1.03 (0.93,1.13)	0.607	1.07 (0.95,1.21)	0.260
	TIV this year	1.03 (0.91,1.16)	0.665	0.94 (0.79,1.11)	0.451
	TIV (-) v.s (+)	1.09 (0.96,1.24)	0.184	1.13 (0.92,1.38)	0.249
	TIV partial v.s (+)	0.91 (0.76,1.09)	0.306	0.85 (0.66,1.08)	0.186
Adults					
**1st Year**	Rural v.s urban	0.98 (0.87,1.21)	0.161	1.02 (0.93,1.12)	0.693
	Household size	1.02 (0.97,1.07)	0.444	1.00 (0.97,1.04)	0.806
	TIV previous year	1.11 (0.91,1.35)	0.314	0.94 (0.90,0.97)	<0.001*
	TIV (-) v.s (+)	1.01 (0.91,1.12)	0.878	0.99 (0.92,1.06)	0.726
**2nd Year**	Rural v.s urban	0.89 (0.82,0.97)	0.006*	0.91 (0.84,0.99)	0.024*
	Household size	0.89 (0.80,1.00)	0.046*	0.99 (0.90,1.08)	0.762
	TIV previous year	0.98 (0.83,1.16)	0.809	1.08 (0.92,1.28)	0.342
	TIV (-) v.s (+)	0.95 (0.85,1.06)	0.359	0.98 (0.89,1.08)	0.688
	TIV partial v.s (+)	1.07 (0.94,1.23)	0.303	1.02 (0.91,1.16)	0.700
**3rd Year**	Rural v.s urban	0.85 (0.75,0.96)	0.010*	0.87 (0.77,0.99)	0.031*
	Household size	1.03 (0.93,1.13)	0.620	1.05 (0.94,1.18)	0.385
	TIV previous year	1.08 (0.91,1.30)	0.381	0.96 (0.80,1.14)	0.627
	TIV (-) v.s (+)	0.94 (0.81,1.09)	0.423	0.95 (0.82,1.10)	0.476
	TIV partial v.s (+)	0.99 (0.83,1.19)	0.919	0.92 (0.75,1.12)	0.402

^$^Odds ratio (95% confidence interval).

*p < 0.05.

Interestingly, after adjusting for households with or without TIV vaccination, lower risk of infection was observed among household adult contacts from the rural residential area compared to those from the urban area (OR = 0.89; 95% CI: 0.82-0.97 for Year 2 and OR = 0.85; 95% CI: 0.75-0.96 for Year 3) (Table 6). Similar results were also observed among adult contacts for infection with clinical symptoms.

## Discussion

Although vaccine recommendations have been gradually expanded to include all persons ≥6 months of age, the vaccination strategy specifically targeting schoolchildren to reduce community-wide or household transmission of seasonal influenza is still debatable [20-22]. Recent school-based and community cluster-randomized control trials suggest that the vaccination of schoolchildren can reduce influenza-related morbidity and mortality among non-immunized contacts within households [14] and in the community [10-13]. Consistent with previous reports, secondary infection risks within households were highest among young contacts [23-25]. In our current study, although no statistically significant reduction of serological-confirmed infection or clinical morbidity among the vaccinated household contacts was observed during three consecutive seasons, a consistent trend was observed among household children contacts from un-vaccinated households having a greater risk of infection or combined clinical morbidity (odds ratio greater than 1) after adjusting for confounding variables. During Year 3, odds ratio less than 1 was observed among children contacts from partial-immunization households, which was probably due to the small sample size. In contrast, no consistent trend was observed among household adult contacts since the resulting odds ratios were not always greater than 1. One reason could be that household adults usually acquire infection from the community [15,26]. In particular, we observed a higher rate of infection in adults living in urban areas than those living in rural areas. Therefore, vaccinating schoolchildren may not provide sufficient protection to the household adult contacts living in urban cities. Since we didn’t measure risk of infection conditioned on the level of exposure, only overall risk was considered in this study by adjusting the confounding variables using GEE methods and the potential bias of household vaccination status might affect the risk of exposure cannot be ruled out; ie. the household contact from unvaccinated households tended to be more careful to prevent having influenza infection. However, higher risk of infection among household adult contacts was observed in the urban area with lower vaccination rate, than rural area with higher vaccination rate (Tables 2 and 6). In the future, large clinical trials considering the transmission intensity in different geographic locations will be needed to assess the benefits of the schoolchildren vaccination policy on the household contacts and community.

Observational studies to assess vaccine effectiveness have become a standard way of routinely evaluating how well influenza vaccines protect population groups in countries implementing influenza vaccination programs [27-30]. These studies usually utilize a sensitive and specific laboratory method such as virus isolation or reverse-transcriptase polymerase chain reaction (RT-PCR) to confirm illness as influenza. These methods may miss a case if administered too early (e.g., during the incubation period) or too late (e.g., after the infectious period) in the disease course. Since many influenza cases are asymptomatic, identifying every case would necessitate serially screening the entire study population throughout the influenza season, which currently would be prohibitively expensive and operationally challenging. The advantage of using serological four-fold increase of HI titers in this study as a surrogate marker of infection was that the true infection rate could be estimated. To measure the true infections among household contacts, all contacts having TIV vaccination records during the contemporary influenza season were pre-excluded from the analysis. The possibility of a delayed rising of antibody titer post-infection, resulting in mis-classification of infection status among different groups, was also considered. The median durations of sera withdrawn were equivalent among household contacts from households with different vaccination statuses. As shown in Table 5, the infection rates from serological results among the household contacts were much higher than those previously published in studies using RT-PCR to estimate infection [12,22,31]; however, consistent with our previous publication [32]. All virus strains used in this study were originated from human, instead of avian-origin, and the negative control was used all the time during performing the assay. Although guinea pig RBC is strongly suggested for HI assay, the potential bias using chicken RBC in this study will be minimal but cannot be completely excluded [33,34]. However, the disadvantage of using serological four-fold increase of HI titers as an infection marker was that if the true benefits of the schoolchildren vaccinating policy is to prevent clinical illness instead of infection, this method under-estimated the vaccination effectiveness. Therefore, no statistically significant reduction of serological-confirmed infection among the vaccinated household contacts was observed during three consecutive seasons in this study due to the high infection rate by influenza virus. The unexpected findings of high infection rate and low effectiveness of household contacts require further study.

Measuring serologically confirmed infection allowed us to compare the transmission and infection of influenza virus in different geographical locations. Our multivariate GEE analysis after adjusting household vaccination status suggested that a lower risk of acquiring infection was observed among household adult contacts from the rural residential area than among those from the urban area with statistical significance (Table 6). Recent studies from 2009 pandemic H1N1 (pH1N1) outbreaks suggested that the transmissions of influenza virus were spatially heterogeneous [15,26]. A different contact profile in different social settings, social and geographic factors probably shape the local reproduction number (R_0_) [35,36]. The crowdedness of urban environments facilitates influenza virus transmission, and adults might acquire infection through variable contact pathways in the community. As such, this might compromise the vaccine effectiveness of the schoolchildren vaccination policy in reducing influenza-related morbidity and mortality of household contacts, particularly in adults. Designing an optimal mitigation measurement tailored to different geographical locations will help to control the influenza epidemic or pandemic.

Another interesting finding in our study was that higher antibody titers against seasonal influenza strains including H1, H3 and B were observed in children who received the pH1N1 vaccine than in those who did not. This observation was independent from whether or not the children received TIV. Previous studies suggested that broadly neutralizing antibodies reactive to the conserved stem region of the influenza virus hemagglutinin (HA) were generated in people infected or vaccinated with the 2009 pandemic H1N1 strain [37,38]. Additionally, other studies also suggested that a vigorous antibody response to pH1N1 vaccination resulted from the activation of preexisting memory B cells, which did not only bind to the stem region but also the head region of HA [39]. Furthermore, cross-reactive T-cell responses can also contribute to the cross-protection. A larger sample size will be needed to confirm this observation.

The main limitation of our study relates to the small sample sizes among subsets of the participants receiving TIV, which reduced both the statistical power and precision. Same reason for the attempt of calculating attack rates within households and compare them among vaccinated, partial and non-vaccinated households. Particularly in Year 2, two vaccines (TIV and pH1N1) were delivered during the same season one month apart. Our previous study demonstrated that children who received TIV prior to pH1N1 had a lower sero-conversion rate than those who received only the pH1N1 vaccine [40]. Whether this was the cause of the higher infection rates among the household contacts observed in our study, as shown in Table 5, requires further confirmation. Potential self-selection bias of study subjects involved in this study is possible, particularly since more families were recruited from rural areas than urban areas in this study. Furthermore, timing of vaccination or period of sera collection coincided with the circulation of influenza viruses might also affect the determination of status of infection, particularly during Year 2. Prior immunity of each individual could also affect the protection level, data of which was available for this study but appeared insignificant due to small sample size after stratification.

## Conclusions

Although drawing a conclusion from data in this observational study requires careful interpretation of the results, due to the possibility of bias of given self-selection for vaccination, assessing the effectiveness of the schoolchildren vaccination policy on protection of household contacts and community would require further research on geographical heterogeneity of influenza transmission and infection.

## Competing interests

We declare that we have no conflict of interest. This work was supported by the National Science Council of Taiwan (NSC 97-2118-M-039-004), China Medical University, Taiwan (CMU 97 323) and Abbott 2012 influenza research grant. The funders had no role in study design, data collection and analysis, decision to publish, or preparation of the manuscript.

## Authors’ contributions

DYC designed the laboratory work and decided the virus strains carried out in this study. CYC and TCL participated in the design of the study, designed the questionnaire and coordinated the training for telephone interview. DYC, KFC, YHH, and TNW conceived of the study, statistical analysis and participated in its design and coordination and helped to draft the manuscript. All authors read and approved the final manuscript.

## Pre-publication history

The pre-publication history for this paper can be accessed here:

http://www.biomedcentral.com/1471-2334/14/369/prepub

## References

[B1] World Health Organization Influenza (Seasonal) http://wwwwhoint/mediacentre/factsheets/fs211/en/indexhtml 2009

[B2] Brown S Tai J Bailey R Cooley P Wheaton W Potter M Voorhees R LeJeune M Grefenstette J Burke D McGlone S Lee B Would school closure for the 2009 H1N1 influenza epidemic have been worth the cost?: a computational simulation of Pennsylvania BMC Public Health 2011 14 353 10.1186/1471-2458-11-353 21599920 PMC3119163

[B3] Iskander M Booy R Lambert S The burden of influenza in children Curr Opin Infect Dis 2007 14 3 259 263 10.1097/QCO.0b013e3280ad4687 17471035

[B4] Fairbrother G Cassedy A Ortega-Sanchez I Szilagyi P Edwards K Molinari N Donauer S Henderson D Ambrose S Kent D Poehling K Weinberg G Griffin M Hall C Finelli L Bridges C Staat M New Vaccine Surveillance Network (NVSN) High costs of influenza: Direct medical costs of influenza disease in young children Vaccine 2010 14 31 4913 4919 10.1016/j.vaccine.2010.05.036 20576536

[B5] Monto A Koopman J Longini I Tecumseh study of illness. XIII. Influenza infection and disease, 1976–1981 Am J Epidemiol 1985 14 6 811 822 4014174 10.1093/oxfordjournals.aje.a114052

[B6] Principi N Esposito S Are we ready for universal influenza vaccination in paediatrics? Lancet Infect Dis 2004 14 2 75 83 10.1016/S1473-3099(04)00926-0 14871631

[B7] Hurwitz E Haber M Chang A Shope T Teo S Giesick J Ginsberg M Cox N Studies of the 1996–1997 inactivated influenza vaccine among children attending day care: immunologic response, protection against infection, and clinical effectiveness J Infect Dis 2000 14 4 1218 1221 10.1086/315820 10979921

[B8] Stevenson E Barrios L Cordell R Delozier D Gorman S Koenig L Odom E Polder J Randolph J Shimabukuro T Singleton C Pandemic influenza planning: addressing the needs of children Am J Public Health 2009 14 Suppl 2 S255 S260 19797738 10.2105/AJPH.2009.159970 PMC4504394

[B9] Fiore A Epperson S Perrotta D Bernstein H Neuzil K Expanding the recommendations for annual influenza vaccination to school-age children in the United States Pediatrics 2012 14 Suppl 2 S54 S62 22383482 10.1542/peds.2011-0737C

[B10] Reichert T Sugaya N Fedson D Glezen W Simonsen L Tashiro M The Japanese experience with vaccinating schoolchildren against influenza N Engl J Med 2001 14 12 889 896 10.1056/NEJM200103223441204 11259722

[B11] Reichert T The Japanese program of vaccination of schoolchildren against influenza: implications for control of the disease Semin Pediatr Infect Dis 2002 14 2 104 111 10.1053/spid.2002.122997 12122948

[B12] Loeb M Russell M Moss L Fonseca K Fox J Earn D Aoki F Horsman G Van Caeseele P Chokani K Vooght M Babiuk L Webby R Walter S Effect of influenza vaccination of children on infection rates in Hutterite communities: a randomized trial JAMA 2010 14 10 943 950 10.1001/jama.2010.250 20215608

[B13] King JJ Stoddard J Gaglani M Moore K Magder L McClure E Rubin J Englund J Neuzil K Effectiveness of school-based influenza vaccination N Engl J Med 2006 14 24 2523 2532 10.1056/NEJMoa055414 17167135

[B14] Hurwitz E Haber M Chang A Shope T Teo S Ginsberg M Waecker N Cox N Effectiveness of influenza vaccination of day care children in reducing influenza-related morbidity among household contacts JAMA 2000 14 13 1677 1682 10.1001/jama.284.13.1677 11015798

[B15] Chowell G Echevarría-Zuno S Viboud C Simonsen L Tamerius J Miller M Borja-Aburto V Characterizing the epidemiology of the 2009 influenza A/H1N1 pandemic in Mexico PLoS Med 2011 14 5 e1000436 10.1371/journal.pmed.1000436 21629683 PMC3101203

[B16] Jian J Chen G Lai C Hsu L Chen P Kuo S Wu H Shih S Genetic and epidemiological analysis of influenza virus epidemics in Taiwan during 2003 to 2006 J Clin Microbiol 2008 14 4 1426 1434 10.1128/JCM.01560-07 18256223 PMC2292966

[B17] Shih S Chen G Yang C Yang W Liu D Lin J Chiu S Chen H Tsao K Huang C Huang Y Mok C Chen C Lin T Wang J Kao C Lin K Chen L Eng H Liu Y Chen P Lin J Wang J Lin C Chan Y Lu J Hsiung C Chen P Su I Laboratory-based surveillance and molecular epidemiology of influenza virus in Taiwan J Clin Microbiol 2005 14 4 1651 1661 10.1128/JCM.43.4.1651-1661.2005 15814980 PMC1081360

[B18] World Health Organization WHO manual on animal influenza diagnosis and surveillance. WHO/CDS/CSR/NCS/20025 2002

[B19] Rowe T Abernathy R Hu-Primmer J Thompson W Lu X Lim W Fukuda K Cox N Katz J Detection of antibody to avian influenza A (H5N1) virus in human serum by using a combination of serologic assays J Clin Microbiol 1999 14 4 937 943 10074505 10.1128/jcm.37.4.937-943.1999 PMC88628

[B20] Centers for Disease Control and Prevention (CDC) Prevention and control of seasonal influenza with vaccines. Recommendations of the Advisory Committee on Immunization Practices--United States, 2013–2014 MMWR Recomm Rep 2013 14 RR-07 1 43 24048214

[B21] Jefferson T Rivetti A Di Pietrantonj C Demicheli V Ferroni E Vaccines for preventing influenza in healthy children Cochrane Database Syst Rev 2012 14 CD004879 22895945 10.1002/14651858.CD004879.pub4 PMC6478137

[B22] Ohmit S Petrie J Malosh R Cowling B Thompson M Shay D Monto A Influenza vaccine effectiveness in the community and the household Clin Infect Dis 2013 14 10 1363 1369 10.1093/cid/cit060 23413420 PMC3693492

[B23] Cowling B Chan K Fang V Lau L So H Fung R Ma E Kwong A Chan C Tsui W Ngai H Chu D Lee P Chiu M Leung G Peiris J Comparative epidemiology of pandemic and seasonal influenza A in households N Engl J Med 2010 14 23 2175 2184 10.1056/NEJMoa0911530 20558368 PMC4070281

[B24] Sugimoto J Borse N Ta M Stockman L Fischer G Yang Y Halloran M Longini IJ Duchin J The effect of age on transmission of 2009 pandemic influenza A (H1N1) in a camp and associated households Epidemiology 2011 14 2 180 187 10.1097/EDE.0b013e3182060ca5 21233714 PMC3755879

[B25] Petrie J Ohmit S Cowling B Johnson E Cross R Malosh R Thompson M Monto A Influenza transmission in a cohort of households with children: 2010–2011 PLoS One 2013 14 9 e75339 10.1371/journal.pone.0075339 24086511 PMC3783407

[B26] Kim C Breiman R Cosmas L Audi A Aura B Bigogo G Njuguna H Lebo E Waiboci L Njenga M Feikin D Katz M Secondary household transmission of 2009 pandemic influenza A (H1N1) virus among an urban and rural population in Kenya, 2009–2010 PLoS One 2012 14 6 e38166 10.1371/journal.pone.0038166 22701610 PMC3372521

[B27] Kissling E Valenciano M Cohen J Oroszi B Barret A Rizzo C Stefanoff P Nunes B Pitigoi D Larrauri A Daviaud I Horvath J O’Donnell J Sevler T Paradowska-Stankiewicz I Pechirra P Ivanciuc A Jimenez-Jorge S Savulescu C Ciancio B Moren A I-MOVE multi-centre case control study 2010–11: overall and stratified estimates of influenza vaccine effectiveness in Europe PLoS One 2011 14 11 e27622 10.1371/journal.pone.0027622 22110695 PMC3216983

[B28] Skowronski D Janjua N De Serres G Hottes T Dickinson J Crowcroft N Kwindt T Tang P Charest H Fonseca K Gubbay J Bastien N Li Y Petric M Effectiveness of AS03 adjuvanted pandemic H1N1 vaccine: case–control evaluation based on sentinel surveillance system in Canada, autumn 2009 BMJ 2011 14 c7297 10.1136/bmj.c7297 21292718 PMC3033439

[B29] Griffin M Monto A Belongia E Treanor J Chen Q Chen J Talbot H Ohmit S Coleman L Lofthus G Petrie J Meece J Hall C Williams J Gargiullo P Berman L Shay D US Flu-VE Network Effectiveness of non-adjuvanted pandemic influenza A vaccines for preventing pandemic influenza acute respiratory illness visits in 4 U.S. communities PLoS One 2011 14 8 e23085 10.1371/journal.pone.0023085 21857999 PMC3155536

[B30] Treanor J Talbot H Ohmit S Coleman L Thompson M Cheng P Petrie J Lofthus G Meece J Williams J Berman L Breese Hall C Monto A Griffin M Belongia E Shay D US Flu-VE Network Effectiveness of seasonal influenza vaccines in the United States during a season with circulation of all three vaccine strains Clin Infect Dis 2012 14 7 951 959 10.1093/cid/cis574 22843783 PMC3657521

[B31] Jackson M France A Hancock K Lu X Veguilla V Sun H Liu F Hadler J Harcourt B Esposito D Zimmerman C Katz J Fry A Schrag S Serologically confirmed household transmission of 2009 pandemic influenza A (H1N1) virus during the first pandemic wave–New York City, April-May 2009 Clin Infect Dis 2011 14 5 455 462 10.1093/cid/cir437 21844028

[B32] Chao D Cheng K Li T Wu T Chen C Tsai C Chen J Chiu H Lu J Su M Liao Y Chan W Hsieh Y Serological evidence of subclinical transmission of the 2009 pandemic H1N1 influenza virus outside of Mexico PLoS One 2011 14 1 e14555 10.1371/journal.pone.0014555 21267441 PMC3022590

[B33] Kayali G Setterquist S Capuano A Myers K Gill J Gray G Testing human sera for antibodies against avian influenza viruses: horse RBC hemagglutination inhibition vs. microneutralization assays J Clin Virol 2008 14 1 73 78 10.1016/j.jcv.2008.04.013 18571465 PMC2574547

[B34] Ampofo W Al Busaidy S Cox N Giovanni M Hay A Huang S Inglis S Katz J Mokhtari-Azad T Peiris M Savy V Sawanpanyalert P Venter M Waddell A Wickramasinghe G Zhang W Ziegler T WHO Writing Group Strengthening the influenza vaccine virus selection and development process: outcome of the 2nd WHO Informal Consultation for Improving Influenza Vaccine Virus Selection held at the Centre International de Conférences (CICG) Geneva, Switzerland, 7 to 9 December 2011 Vaccine 2013 14 32 3209 3221 23685246 10.1016/j.vaccine.2013.05.049

[B35] Valleron A Cori A Valtat S Meurisse S Carrat F Boëlle P Transmissibility and geographic spread of the 1889 influenza pandemic Proc Natl Acad Sci U S A 2010 14 19 8778 8781 10.1073/pnas.1000886107 20421481 PMC2889325

[B36] Kretzschmar M Mikolajczyk R Contact profiles in eight European countries and implications for modelling the spread of airborne infectious diseases PLoS One 2009 14 6 e5931 10.1371/journal.pone.0005931 19536278 PMC2691957

[B37] Li G Chiu C Wrammert J McCausland M Andrews S Zheng N Lee J Huang M Qu X Edupuganti S Mulligan M Das S Yewdell J Mehta A Wilson P Ahmed R Pandemic H1N1 influenza vaccine induces a recall response in humans that favors broadly cross-reactive memory B cells Proc Natl Acad Sci U S A 2012 14 23 9047 9052 10.1073/pnas.1118979109 22615367 PMC3384143

[B38] Wrammert J Koutsonanos D Li G Edupuganti S Sui J Morrissey M McCausland M Skountzou I Hornig M Lipkin W Mehta A Razavi B Del Rio C Zheng N Lee J Huang M Ali Z Kaur K Andrews S Amara R Wang Y Das S O’Donnell C Yewdell J Subbarao K Marasco W Mulligan M Compans R Ahmed R Wilson P Broadly cross-reactive antibodies dominate the human B cell response against 2009 pandemic H1N1 influenza virus infection J Exp Med 2011 14 1 181 193 10.1084/jem.20101352 21220454 PMC3023136

[B39] Sangster M Baer J Santiago F Fitzgerald T Ilyushina N Sundararajan A Henn A Krammer F Yang H Luke C Zand M Wright P Treanor J Topham D Subbarao K B cell response and hemagglutinin stalk-reactive antibody production in different age cohorts following 2009 H1N1 influenza virus vaccination Clin Vaccine Immunol 2013 14 6 867 876 10.1128/CVI.00735-12 23576673 PMC3675965

[B40] Chao D Cheng K Hsieh Y Li T Wu T Chen C Tsai C Chen J Chiu H Lu J Su M Liao Y CIDER Serological response and persistence in schoolchildren with high baseline seropositive rate after receiving 2009 pandemic influenza A(H1N1) vaccine Vaccine 2011 14 4 617 623 10.1016/j.vaccine.2010.11.016 21095255

